# Supplementary material for: Revealing the Altermagnetism in Hematite via XMCD Imaging and Anomalous Hall Electrical Transport
Source: Adv Mater. 2025 Jul 29;37(41):e05019. doi: 10.1002/adma.202505019 (PMC12531726; doi:10.1002/adma.202505019)
Supplement: Supplementary file 1 — Supporting Information [file ADMA-37-e05019-s001.pdf]

ADVANCED MATERIALS

Supporting Information

for *Adv. Mater.*, DOI 10.1002/adma.202505019

Revealing the Altermagnetism in Hematite via XMCD Imaging and Anomalous Hall Electrical Transport

*Edgar Galindez-Ruales, Rafael Gonzalez-Hernandez, Christin Schmitt, Shubhankar Das, Felix Fuhrmann, Andrew Ross, Evangelos Golias, Akashdeep Akashdeep, Laura Lünenbürger, Eunchong Baek, Wanting Yang, Libor Šmejkal, Venkata Krishna, Rodrigo Jaeschke-Ubiergo, Jairo Sinova, Avner Rothschild, Chun-Yeol You, Gerhard Jakob and Mathias Kläui**

Revealing the Altermagnetism in Hematite via XMCD Imaging and Anomalous Hall Electrical Transport: Supporting Information

Edgar Galindez-Ruales,[1], Rafael Gonzalez-Hernandez[2,1†], Christin Schmitt[1†], Shubhankar Das[1], Felix Fuhrmann[1], Andrew Ross[1], Evangelos Golias[3], Akashdeep Akashdeep[1], Laura Lünenbürger[1], Eunchong Baek[4,1], Wanting Yang[1], Venkata Krishna[1], Rodrigo Jaeschke-Ubriego[1], Libor Šmejkal[5,6,7,1], Jairo Sinova[1], Avner Rothschild[8], Chun-Yeol You[4], Gerhard Jakob[1], Mathias Kläui[1,9*]

1 Institute of Physics, Johannes Gutenberg University Mainz, Mainz, Germany.

2 Grupo de Investigacíon en Física Aplicada, Departamento de Física, Universidad del Norte, Barranquilla, Colombia.

3 MAX IV Laboratory, Lund, Sweeden.

4 Department of Physics and Chemistry, DGIST, Daegu, Korea (the Republic of).

5 Max Planck Institute for the Physics of Complex Systems, Nöthnitzer Str. 38, Dresden, Germany.

6 Max Planck Institute for Chemical Physics of Solids, Nöthnitzer Str. 40, Dresden, Germany.

7 Institute of Physics, Academy of Sciences of the Czech Republic, Prague, Czechia.

8 Department of Materials Science and Engineering, Technion-Israel Institute of Technology, Haifa, Israel.

9 Center for Quantum Spintronics, Norwegian University of Science and Technology, Trondheim, Norway.

* klaeui@uni-mainz.de

Transverse voltage symmetrization

Figure S1 shows a polar plot of the symmetric (field-even) and asymmetric (field-odd) components of the transverse conductivity under an out-of-plane magnetic field of 11 T. The symmetric contribution, σ_{xy}^{S} (panel a), exhibits a clear 4-fold symmetry with maximum amplitude at orientations deviating from the patterning axes (along **x** and **y**). This signal is attributed to the magnetoresistance component (AMR), either the longitudinal component leaking into the transverse channel due to the slight misalignment of the Hall bar electrodes, or the projection of the Néel vector into the current channel (PHE).

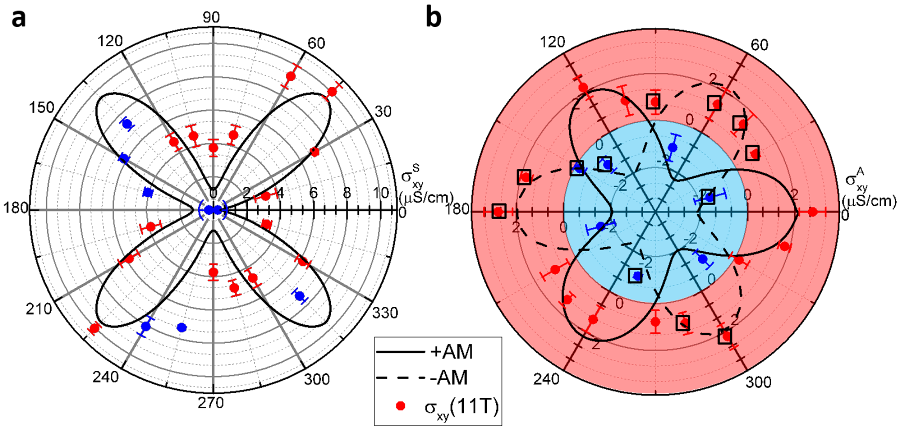

Figure S1: Polar plot of the symmetric and antisymmetric AHC under an 11 T OOP field. (**a**) The symmetric part, $\sigma_x^S y$, shows an increment on the amplitude when the Hall bars deviated from the patterning axis **x** and **y**, and follows a 4-fold symmetry (black line) in amplitude, red (blue) data positive (negative). In the asymmetric part, $\sigma_x^A y$ (**b**), overlaid with two possible 6-node altermagnetic order parameters: positive AM (continuous line) and negative AM (dashed line, for squared symbols) . Here, additional data from a 180°-rotated Hall bar are included, demonstrating that a rotated Hall bar is equivalent to the original due to Hall bar geometry. The 180°-rotated data correspond to an altermagnetic order parameter with opposite sign, consistent with the nodal plane characteristics of G-wave altermagnets.

However, a sign change can more easily occur here when the transverse

voltage probes are not perfectly orthogonal to the current path, leading to partial pickup of the longitudinal resistance with a different sign. Importantly, we exclude planar Hall effect (PHE) contributions to this signal, as a β-scan preceded each measurement to ensure that the magnetisation was aligned along the out-of-plane direction with an angular uncertainty below 0.5°, which suppresses the in-plane component required for PHE. Therefore, the observed 4-fold symmetric pattern in the even signal is not intrinsic but arises from geometric misalignment and is unrelated to the anomalous Hall conductivity (AHC) discussed in the main text.

Due to the intrinsic geometric symmetry of the Hall bar, a 180°-rotated Hall bar is equivalent to the original, and thus, the data has the same sign but is located in opposite extremes of the polar plot. However, this rotated dataset represents an altermagnetic order parameter with an opposite sign, a Hallmark of the symmetry properties inherent to G-wave altermagnets. The six nodal points of both order parameters (magenta and black lines in Figure S1.b) overlap and correspond to the predicted symmetry-driven sign changes in the AHC, which emerge from the interplay between the Néel vector orientation and the crystal symmetry. This dual visualization, through hysteresis and polar plots, emphasizes the critical role of geometry and symmetry in understanding the unique transport properties of altermagnets. These results provide a clear experimental signature of the nodal plane characteristics and symmetry-induced sign inversions that define G-wave altermagnetic systems.

Along less symmetrical orientations (e.g., 45°), the odd contributions are reduced while strong transverse conductivities are present with respect to the field direction. These non-Hall-like contributions stem from symmetry reduction due to the relative orientation of the injected current and the

conductivity tensor and even show a clear sign change (black lines on the left and right panels in Figure S2). A non-odd signal can typically arise from trivial sources, such as misalignment in the Hall bar. This even transverse voltage has a nontrivial origin in our case, as the correlation in the sign inversion and their presence is consistent for multiple different devices (all fabricated in the same lithography and etching steps). We point, as a source of this non-Hall signal, to asymmetries in the conductivity tensor that are only accessible when the anomalous Hall vector allows for the measurement of a transverse voltage, and the symmetry of the system is lowered when the current is flowing in a non-high-symmetry direction. To extract the Hall-like

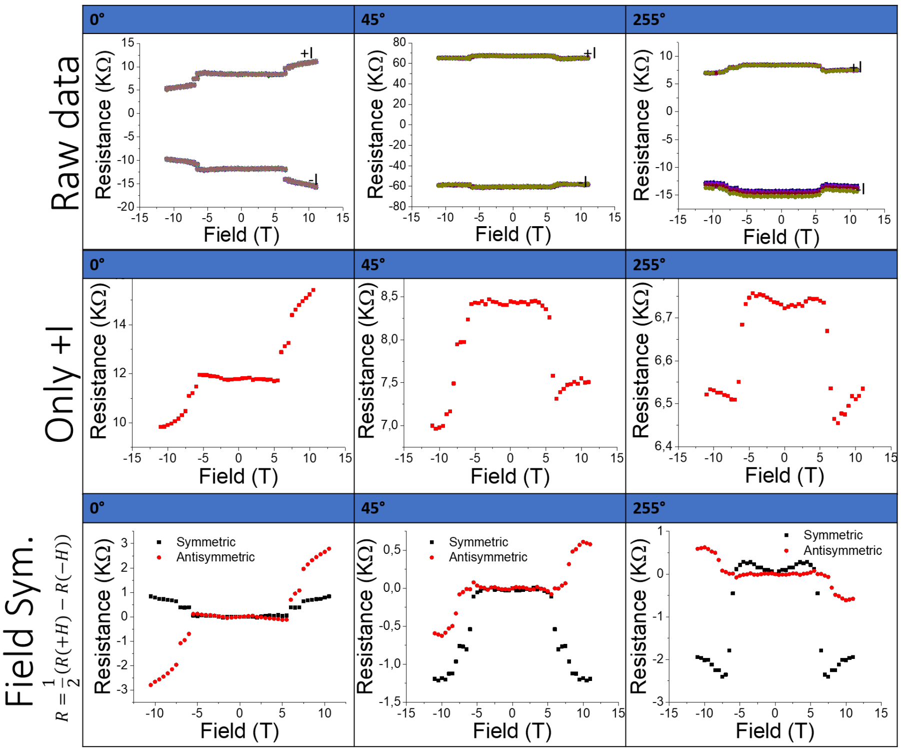

Figure S2: Transversal resistance as a function of the magnetic field in different parts of the field symmetrization process. Raw data (20 cycles), positive Hall, and symmetric (minus the average zero field offset) and antisymmetric part of the resistance for the Hall bars around 0°, 45°, and 255°.

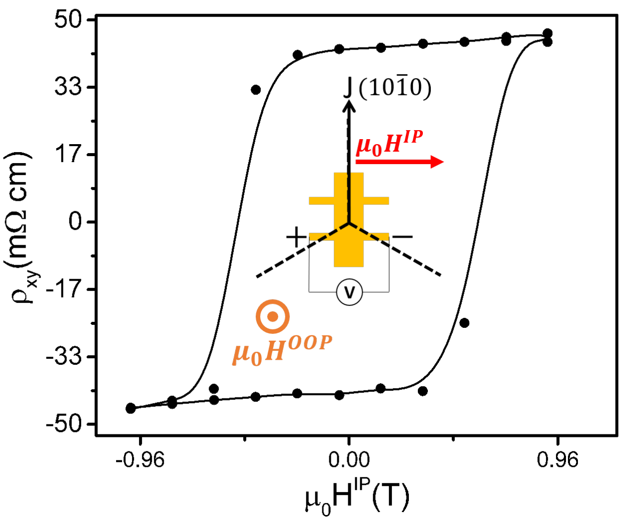

Figure S3: Hysteresis curve of the transverse resistivity as a function of an IP magnetic field angle (perpendicular to the Hall bar), measured with a 10 T OOP magnetic field in the easy-axis phase (300 K). The OOP field ensures the system remains above the spin-flop transition, where the AHC is finite. A schematic of the Hall bar and the applied magnetic fields is presented.

signal from other contributions, the transverse contribution (in our local coordinated system) is symmetrized/antisymmetrized with respect to the external magnetic field using the equation:

$$2\sigma_{xy}^{A(S)} = \sigma_{xy}(H) - (+)\sigma_{xy}(-H) \tag{1}$$

The symmetrization process is illustrated in Figure S2, where the transverse full signal and the even and odd components are presented. According to the theory calculations, a 180° rotation of the Néel vector implies an inversion of sign in the anomalous Hall conductivity (AHC). Figure S3.a illustrates the transverse resistivity hysteresis as a function of the in-plane (IP) magnetic field angle, measured while maintaining a strong 10 T out-of-plane (OOP) magnetic field. This OOP field ensures the system remains above the

spin-flop transition, stabilizing the easy-plane phase where the AHE is finite. In this configuration, the application of an IP magnetic field perpendicular to the Hall bar induces a 180° rotation of the Néel vector. This rotation directly leads to a sign inversion in the Hall conductivity, which matches the theoretical predictions for altermagnetic systems. The hysteretic behavior further demonstrates the robust coupling between the Néel vector orientation and the AHC contributions, providing direct evidence for the angle-dependent symmetry breaking that characterizes altermagnetic transport.

In our measurement scheme, a current density $\mathbf{J} = (J_x, J_y, 0)$ confined to the x-y plane can be expressed as:

$$\mathbf{J} = J_A(\cos\phi, \sin\phi, 0), \tag{2}$$

where J_A is the current amplitude and ϕ is the angle between the direction of the current and the crystallographic a axis, which is aligned with the x axis. The electric field $\mathbf{E}$ produced by this current, considering the conductivity tensor σ_{ij}, is given by:

$$\mathbf{J} = \sigma_{ij}\mathbf{E}. \tag{3}$$

In a conventional ferromagnet, the anomalous Hall vector is proportional to magnetization, and an in-plane rotation of the current under an out-of-plane (OOP) magnetic field would yield no change in the transverse voltage since the conductivity tensor σ_{ij} is invariant under rotations around the symmetry axis. However, in our altermagnetic system, the non-diagonal conductivity components depend strongly on the orientation of the Néel vector. While an external OOP magnetic field would yield a constant contribution related to the canted magnetic moment, the non-zero components σ_{xz} and σ_{yz} introduce a ϕ-dependent transverse electric field $\mathbf{E}$, even with constant OOP

magnetization. Writing explicitly the tensor equations (with Hall symmetry ($\sigma_{xy} = -\sigma_{yx}$), we have:

$$\begin{aligned} \sigma_{xx} E_x &+ \sigma_{xy} E_y + \sigma_{xz} E_z = J_A \cos\phi, \\ -\sigma_{xy} E_x &+ \sigma_{yy} E_y + \sigma_{yz} E_z = J_A \sin\phi, \\ -\sigma_{xz} E_x &- \sigma_{yz} E_y + \sigma_{zz} E_z = 0. \end{aligned} \tag{4}$$

Assuming for simplicity J_A =1 A/m^2, and an isotropic longitudinal conductivity ($\sigma_{xx} = \sigma_{yy} = \sigma_{zz}$), the perpendicular electrical field, defined as $E_\perp = E_x \cdot \sin(\phi) + E_y \cdot \cos(\phi)$, takes the form:

$$E_\perp = \frac{\sigma_{xx}^2 \sin(2\phi) + \sigma_{xx}\sigma_{xy} \cos(2\phi) + \frac{1}{2}\sigma_{xz}^2 \sin(2\phi) - \sigma_{xz}\sigma_{yz} + \frac{1}{2}\sigma_{yz}^2 \sin(2\phi)}{\sigma_{xx}\left(\sigma_{xx}^2 + \sigma_{xy}^2 + \sigma_{xz}^2 + \sigma_{yz}^2\right).} \tag{5}$$

Similarly, the longitudinal component, defined as $E_\parallel = E_x \cdot \cos(\phi) - E_y \cdot \sin(\phi)$, is:

$$E_\parallel = \frac{\sigma_{xx}^2 \cos(2\phi) - \sigma_{xx}\sigma_{xy} \sin(2\phi) + \frac{1}{2}\sigma_{xz}^2 (\cos(2\phi) - 1) + \frac{1}{2}\sigma_{yz}^2 (\cos(2\phi) + 1)}{\sigma_{xx}\left(\sigma_{xx}^2 + \sigma_{xy}^2 + \sigma_{xz}^2 + \sigma_{yz}^2\right).} \tag{6}$$

The resulting electric field inherits its angular dependence from the Néel-vector-sensitive conductivity tensor components σ_{ij} (which depends on the Energy $E - E_f = -0.12eV$, in our calculations). This dependence, plotted as a function of the current orientation ϕ and Néel vector angle relative to the **a**-axis, is shown in Fig. S4. The exact form of this angular dependence is dictated by the relative magnitudes of the tensor components σ_{ij}, which themselves depend on the electronic structure and Fermi level positioning in the conduction bands. Our measured Hall voltage is then a convolution of the AHC that depends on the Néel vector orientation with the crystallographic

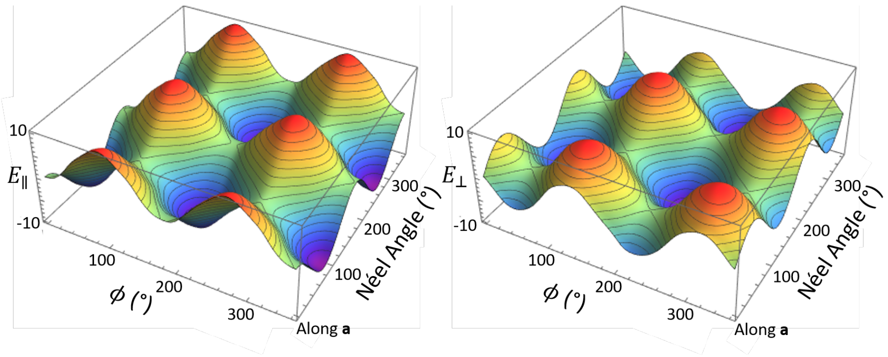

Figure S4: Surface plots of the calculated longitudinal (**a**) and transverse (**b**) electric field components as functions of Hall-bar orientation angle (ϕ) and Néel (vector) angle, relative to the crystallographic axis **a**. Both field components exhibit a clear angular dependence, including periodic sign inversions. The transverse anomalous Hall conductivity (AHC) reflects a convolution of the intrinsic AHC tensor symmetry (which features nontrivial in-plane and out-of-plane components, as shown in the main text) and the Hall-bar geometry (with equivalent signals at 0° and 180° orientations). This analysis emphasizes that the anomalous Hall vector is not simply perpendicular to the Néel vector or strictly oriented along the out-of-plane direction but displays a more complex angular dependence.

axis, a contribution from the AHE from the canted moment, and the lowering of the symmetry induced by the patterning of the Hall bars.

In total, 24 different directions ϕ were measured, Fig. S5 shows the average σ_{xy} of all the different devices, the asymmetric and the symmetric part.

The electrical measurements were performed in a ^{4}He cryostat, capable of temperatures between 1.6 K and 350 K and magnetic fields up to 12 T. Thermal stabilization was ensured during all the measurements, which were monitored by a Cernox sensor element with a percentual relative variation of less than 0.03%. The tendency lines were calculated based on B-spline interpolation, and the dispersion across twenty magnetic field cycles on each device was used to calculate the errors.

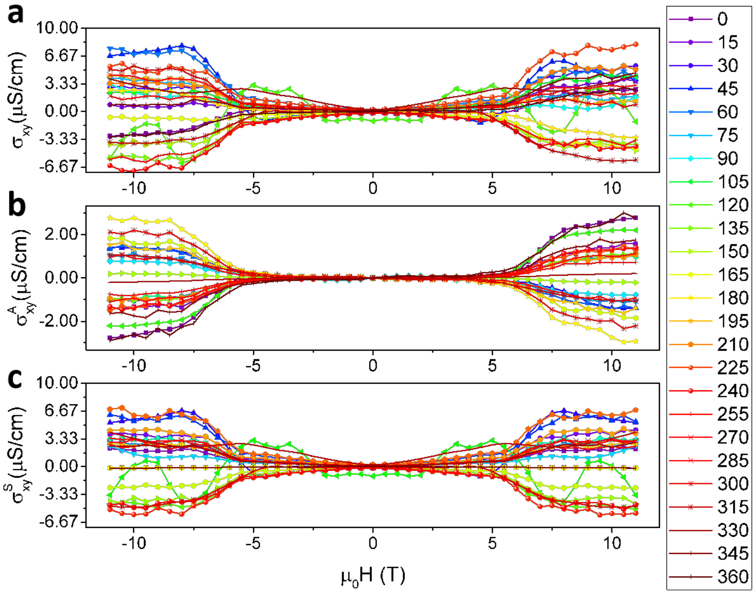

Figure S5: Transverse conductivity σ_{xy} as a function of out-of-plane magnetic field for 24devices patterned at different in-plane angles ϕ, ranging from 0° to 360° in 15° increments. **a** The total signal σ_{xy} is separated into its **b** antisymmetric (odd-in-field) component, and **c** symmetric (even-in-field) contribution. Each trace corresponds to a different device orientation, color-coded according to the legend. These measurements form the basis for the angular analysis of the anomalous Hall response and the identification of contributions unrelated to the Hall signal (e.g., from AMR leaking to the signal due to misalignment).

Geometry for X-ray Photoemission electron microscopy (XPEEM) imaging in hematite

The schematic in Figure S6 depicts the experimental geometry employed for X-ray circular dichroism (XMCD) and linear dichroism (XMLD) measurements in hematite using XPEEM. The sample is oriented along the R-cut crystallographic plane, enabling precise probing of its magnetic properties. The incident X-ray beam, represented by the dashed magenta line, interacts

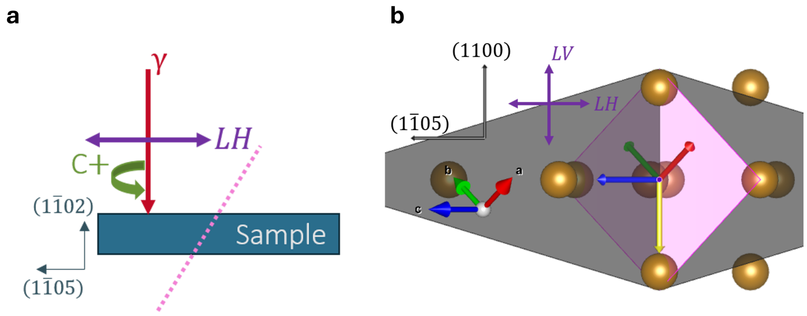

Figure S6: Schematic representation of the experimental geometry for XMLD and XMCD imaging in hematite. **a** The sample, oriented along the R-cut crystal plane, is illuminated with X-rays in linear horizontal (LH), linear vertical (LV), and circular polarization (C+). The coordinate system of the sample is defined relative to its crystallographic axes, with the incident beam direction indicated by the magenta dashed line. **b** Visualization of the local crystallographic structure of hematite, showing the oxygen octahedra surrounding iron atoms and the alignment of the crystal axes. The orientation of the Néel vector relative to the X-ray polarization enables magnetic domain mapping and symmetry analysis via XMLD and XMCD.

with the sample at an oblique angle, providing sensitivity to both the IP and OOP magnetic components. Linear horizontal (LH) and linear vertical (LV) polarizations are used for XMLD measurements to map the IP Néel vector direction, while circularly polarized X-rays (C±) are utilized for XMCD imaging to detect the weak ferromagnetic moment or spin canting caused by symmetry-breaking effects. The local crystallographic structure, shown on the right, highlights the alignment of the crystal axes and the oxygen octahedra surrounding the iron atoms, which are essential for understanding the altermagnetic band structure. This experimental setup allows for direct visualization of the Néel vector and symmetry-related magnetic properties, bridging the gap between real-space imaging and transport measurements in altermagnetic hematite. Figure S7 presents the XAS data corroborating the XMCD and XMLD contrast mechanisms used in the XPEEM imaging experiments. The XMCD spectrum **a** reveals clear dichroic features at the

Fe L_3 edge, indicative of a net magnetization along the X-ray propagation direction, which in our setup corresponds to the out-of-plane component. Meanwhile, the XMLD spectrum **b** shows a distinct polarization-dependent absorption response, confirming sensitivity to the Néel vector orientation. These spectra validate the application of XMCD and XMLD contrast to map the altermagnetic domain structure of hematite.

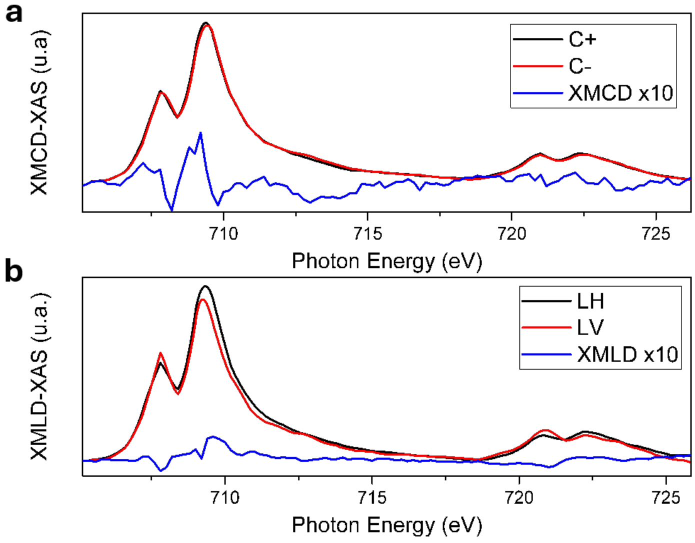

Figure S7: X-ray absorption spectroscopy (XAS) measurements for hematite. **a** XAS spectra acquired using circularly polarized light (C^+ and C^-) showing the Fe $L_{2,3}$ absorption edges, with the corresponding XMCD signal (C^+˘C^-) displayed in blue and amplified by a factor of 10. **b** XAS spectra taken with linearly polarized light in horizontal (LH) and vertical (LV) configurations, with the resulting XMLD (LH˘LV), also magnified x10 for clarity.

Figure S8 presents the XMLD results for Ti-doped hematite. The micrograph in panel **a** reveals clear magnetic domain contrast, demonstrating that XMLD remains a viable contrast mechanism even with Ti doping and has sizable magnetic domains. The XAS spectra in panel **b** show a polarization-dependent absorption at the Fe L_2 edge, and the resulting XMLD signal (green) confirms the sensitivity to Néel vector orientation. This supports the

claim that the altermagnetic symmetry is preserved in the doped system. In the MAXPEEM setup, the incident X-ray beam arrives perpendicular to

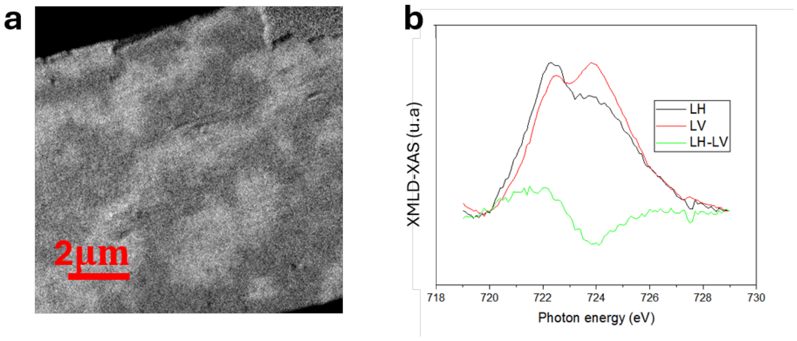

Figure S8: XMLD characterization of Ti-doped hematite. **a.** XPEEM micrograph obtained using linearly polarized light at the Fe L_2 edge, showing magnetic contrast due to XMLD. The domain structure exhibits micrometer-scale contrast, revealing regions with distinct Néel vector orientations. **b.** X-ray absorption spectra (XAS) acquired with linear horizontal (LH) and vertical (LV) polarizations, along with their difference (LH˘LV, green), confirming the XMLD signal used for imaging.

the surface plane. For XMCD contrast to be detected, the projection of the anomalous Hall vector along the beam direction must be significant.

In our Ti-doped hematite sample, the crystal is oriented such that the [0001] axis is normal to the surface. This means that any out-of-plane magnetic components (including the canted moments responsible for XMCD contrast) lie parallel to the beam direction only if they point significantly out of the surface plane. However, in the easy-plane phase of hematite (above the Morin transition), the net magnetic moment from canting lies in the sample plane (**a-c** plane), nearly perpendicular to the incoming beam, as well as the anomalous Hall vector. As a result, its projection onto the beam axis is minimal.

This unfavorable geometry severely suppresses the XMCD signal, making XMCD visualization in our configuration unfeasible. Therefore, our magnetic

imaging relies entirely on XMLD, which is sensitive to only Néel vector orientation.

Crystalline and epitaxial quality determination

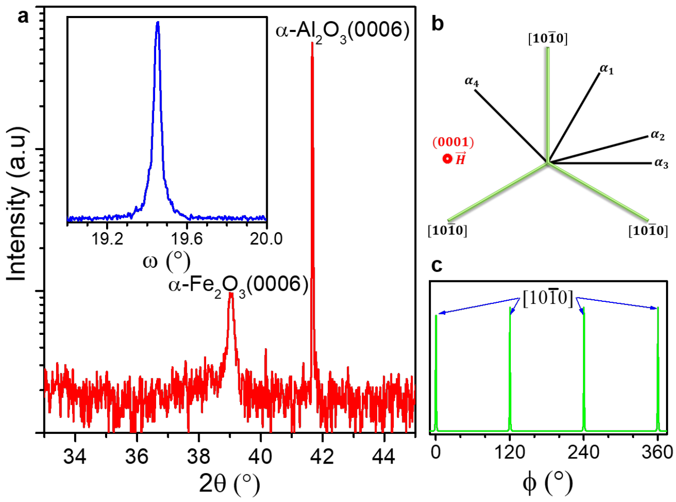

Figure S9: **a** X-ray diffraction analysis of Ti-doped α-Fe_2O_3 with an (0001) orientation did not reveal any secondary phases. The very narrow rocking curve of the (0006) peak in the inset shows epitaxial growth. **b** The diagram illustrates the 3-fold symmetry of the crystallographic plane. Here, the α_2 and α_4 angles are at the same distance as the IP high symmetry axis but in different directions, as are the α_1 and α_3. **c** The IP XRD scan shows the crystal's symmetry, with a 120-degree separation between equivalent reflections of the [10$\bar{1}$4], interpreted as projections of the **a**-axis.

X-ray measurements were carried out on the thin-film samples before and after the etching process using a four-circle Bruker D8 Discover thin-film diffractometer. $\theta/2\theta$ measurements (Figure S9.a) show the diffraction from highly oriented Ti-doped hematite films without any impurity phase. ϕ-scans (Figure S9.c) on the (10$\bar{1}$2) peak allowed us to identify the orientations of the [100] IP directions. This information was used to pattern Hall bars in

different orientations with respect to the crystallographic direction **a**.

Figure S9.a shows the XRD pattern, where a diffraction peak at $2\theta = 39°$ is observed, an angle slightly smaller than that for the bulk material [1]. This finding confirms previous reports on homogeneous doping of hematite grown by PLD [2]. The (0001)-orientation of the films was further confirmed by ω scans of the (0006) peak (inset in Figure S9.a). The lattice parameters were calculated from the peak positions and found to reproduce the thin film literature values of a = 5.05(1) Å and c = 13.72(2) Å [3].

Magnetic characterization of the doped samples

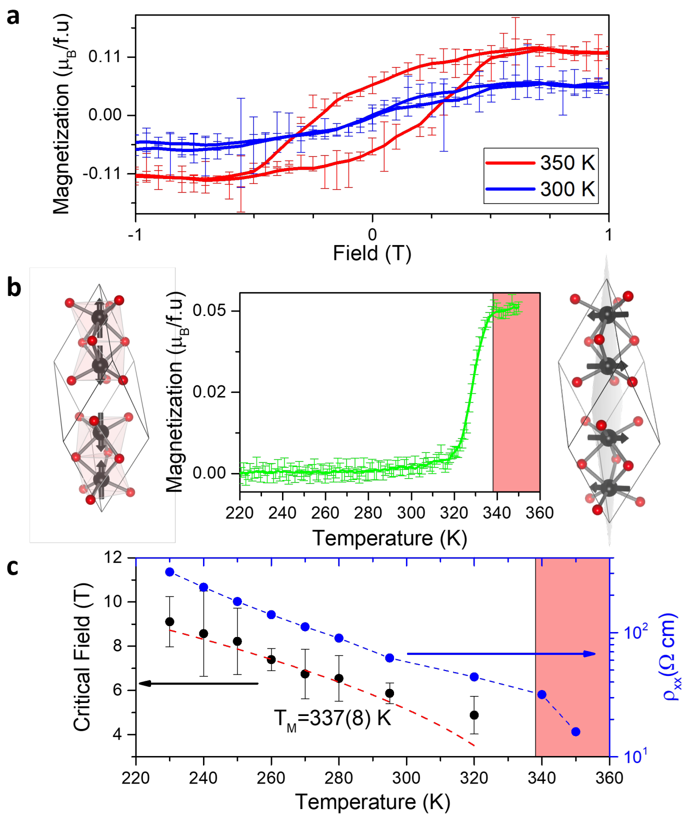

Figure S10: SQUID magnetometry measurements for a magnetic field applied perpendicular to the c-axis. **a** Magnetization per formula unit as a function of the magnetic field for the Ti-doped samples in the collinear phase (300 K) and the weak ferromagnetic phase (350 K). **b** The magnetization as a function of temperature under an external magnetic field of 20 mT reveals an increase in magnetization as temperature increases due to the Morin transition. This transition, from the collinear phase (left panel) to the weak ferromagnetic phase (right panel), occurs over a temperature range of 20 Kelvin. **c** The critical field (H_c) required to reach the spin flop state varies depending on the temperature, while the longitudinal resistivity decreases proportionally. The error bars represent the standard error of the measurement **a** and **b** or the fitting **c**.

The magnetometry measurements (Figure S10) were performed using a commercial superconducting quantum interference device (SQUID) from Quantum Design. The films were saturated with 5 T at a starting temperature of 355 K and then cooled at a constant rate of 2 K/min under a static applied field of $\mu_0 H = 20$ mT. Due to the diamagnetic contribution to the signal from the sapphire substrates used to grow the films and the mounting of the sample in the SQUID, a temperature-independent subtraction of the signal was made from the data from the average value of the magnetization below the observed transition for the temperature scans and, as a linear subtraction calculated at higher fields (± 3 T to $\pm$ 5 T) for the field scans.

The Morin transition of the films was observed and characterized by a decrease in the magnetic moment to effectively zero above the background substrate contributions. The transition occurs at approximately 330 K. At 320 K, the transition was complete, indicating that the films were in the purely easy-axis phase below that temperature. The SQUID measurements agree with other reports, where dopants were found to increase the Morin transition temperature even above room temperature [4]. The presence of Ti atoms did not significantly affect the magnetic moments in the samples, as the magnitude of the canted moment in the weak ferromagnetic phase (WFM) phase falls within the range reported for pure single crystals [1, 5]. Both the temperature increase and the broadening of the Morin transition can be attributed to the smaller unit cell of the film compared to the bulk values, leading to a change in the temperature dependence of the competing anisotropies that underlie the physics of the Morin transition [6].

The SQUID magnetometry measurements provide critical insights into the magnetic behavior of Ti-doped hematite, specifically highlighting the effects of the Morin transition and the weak ferromagnetic phase. At low

temperatures, the samples exhibit a collinear antiferromagnetic order, characterized by negligible magnetization in response to the applied field. As the temperature increases beyond the Morin transition, the system enters the weak ferromagnetic phase, where a canted magnetic moment emerges due to spin reorientation. This is evident in the hysteresis loops, where the magnetic signal saturates at approximately 0.5 T at 350 K, which is in agreement with the reported bulk properties of hematite. At elevated temperatures, the hysteresis loop for the canted moment saturates at ~ 0.5 T. The small signal observed at lower temperatures is attributed to the nucleation of the γ-Fe_2O_3 phase at the substrate interface [4, 7]. The observed magnetic behavior is consistent with the expected characteristics of hematite, indicating that the 1% Ti doping level does not significantly alter the intrinsic magnetic properties of the material. The Morin transition, observed over a temperature range of approximately 20 K, coincides with an increase in magnetization under a low external magnetic field, further corroborating the magnetic phase transition. The temperature dependence of the transverse resistivity follows a square root relation ($H_{cr}(T) \propto \sqrt{T_c - T}$), as predicted by Landau's theory, and fitting better (r^2 0.94 vs. 0.92) than a linear relation.. In contrast, the proportional decrease in longitudinal resistivity reflects the role of hopping transport within the impurity band.

Transport properties in the weak ferromagnetic phase

The Hall conductivity shows a dependence on the relative crystal orientation in the weak ferromagnetic phase and exhibits a change in sign between different orientations of the Hall bars. These aspects do not appear at lower fields in the easy axis phase and are not only contributions from the canted moment.

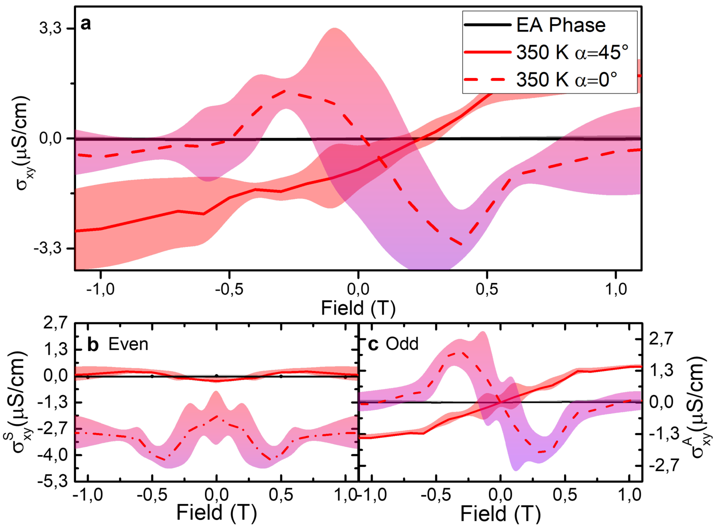

Figure S11: **a** The Hall conductivities of devices along the easy-axis phase (**a**-axis) were measured and compared with the conductivities in the WFM phase and at a -45-degree relative orientation. The even **b** and odd **c** parts of the signal reveal a sign inversion in the odd contribution with the two relative orientations.

In the high-temperature regime, the altermagnetic nature of hematite remains unchanged, even in the presence of a finite canted moment. Here, a reorientation of the Neel order and the saturation of the canted moment require a small magnetic field (as shown in the main text). In the Hall-like signal (Figure S11), in the sample along the high-symmetry crystalline axis, a typical AHE signal is observed, with a saturation field that is consistent with the SQUID measurement results. However, the signal of the Hall bar that is oriented in the "altermagnetic direction" is not proportional to the canted moment. In this case, a competition between an AHE coming from the canted moment and the signal arising from the altermagnetic nature, which is opposite in sign, are competing and result in a saturation of around 0 for positive and negative fields.

In the low-temperature regime, when the Néel vector aligns along the OOP easy axis, consistent with the phase below the Morin transition, we

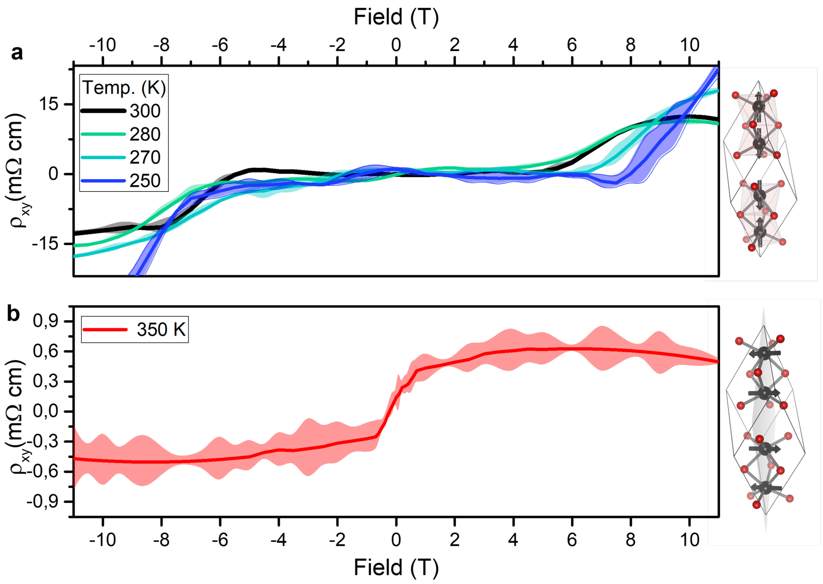

Figure S12: Hall resistivity in relation to the OOP field for temperatures both below **a** and above **b** the Morin transition. The shadow color denotes the standard deviation across 20 different field cycles. The shift in the critical field with temperature in the collinear phase signifies the correspondence between the spin-flop and the Morin transition, as expected for hematite. The magnetic configurations of the iron (Fe) atoms in both phases are depicted on the right side of the graph.

observe no significant AHE, nether in the electrical measurements (below the spin flop) nor in the XMCD imaging, as supported by our DFT calculations (Figure **??**). This absence of AHC correlates with the lack of an electrical signal in transport measurements and the lack of a detectable XMCD signal, which both confirm the absence of a net spin polarization or symmetry breaking required for finite AHC. In contrast, in the high-temperature regime, where the Néel vector prefers the IP easy plane orientation, finite AHC contributions emerge, consistent with symmetry-allowed mechanisms in the canted antiferromagnetic phase. These findings validate the theoretical predictions and highlight the critical role of Néel vector orientation in determining the transport properties of hematite.

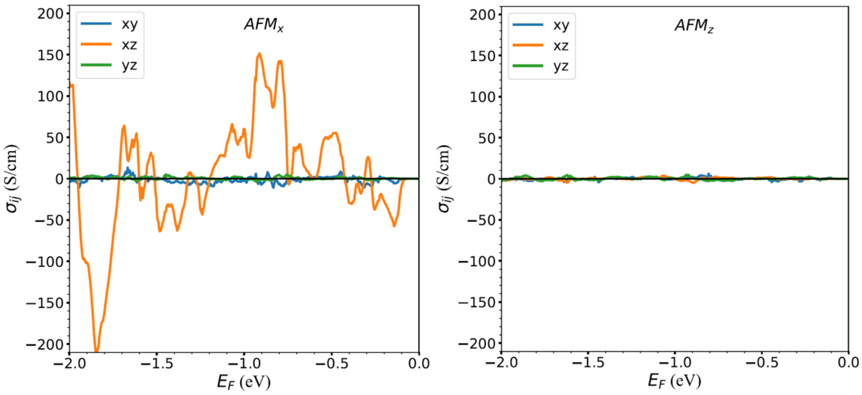

Figure S13: The reorientation of the Néel vector across the Morin transition changes the AHC contributions. **a** In the high-temperature regime, the Néel vector prefers to point along the IP easy-axis (**a**-axis) and has a finite anomalus Hall conductivity. **b** When the Néel vector aligns with the OOP easy-axis below the Morin transition, we observe no significant contribution.

References

[1] A.H. Morrish. *Canted Antiferromagnetism: Hematite*. World Scientific, Jan. 1994. ISBN: 9789810220075.

[2] K. D. Malviya et al. "Systematic comparison of different dopants in thin film hematite (α-Fe_2O_3) photoanodes for solar water splitting". In: *J. Mater. Chem. A* 4 (8 Dec. 2016), pp. 3091–3099. DOI: 10.1039/C5TA07095C.

[3] O. M. Lemine et al. "Rietveld analysis and Mössbauer spectroscopy studies of nanocrystalline hematite α-Fe_2O_3". In: *J. Alloys Compd.* 502.2 (July 2010), pp. 279–282. ISSN: 0925-8388. DOI: https://doi.org/10.1016/j.jallcom.2010.04.175.

[4] A. Ross et al. "An insulating doped antiferromagnet with low magnetic symmetry as a room temperature spin conduit". In: *Appl. Phys. Lett.* 117.24 (Dec. 2020), p. 242405. DOI: 10.1063/5.0032940.

[5] R. Lebrun et al. "Tunable long-distance spin transport in a crystalline antiferromagnetic iron oxide". In: *Nature* 561.7722 (2018), 222–225. DOI: 10.1038/s41586-018-0490-7.

[6] J. O. Artman, J. C. Murphy, and S. Foner. "Magnetic Anisotropy in Antiferromagnetic Corundum-Type Sesquioxides". In: *Phys. Rev.* 138 (3A May 1965), A912–A917. DOI: 10.1103/PhysRev.138.A912.

[7] I. Dzyaloshinsky. "A thermodynamic theory of "weak" ferromagnetism of antiferromagnetics". In: *J. Phys. Chem. Solids* 4.4 (Aug. 1958), pp. 241–255. ISSN: 0022-3697. DOI: https://doi.org/10.1016/0022-3697(58)90076-3.
